# Dose-response association of sleep quality with anxiety symptoms in Chinese rural population: the Henan rural cohort

**DOI:** 10.1186/s12889-020-09400-2

**Published:** 2020-08-27

**Authors:** Jiali Shen, Haiqing Zhang, Yan Wang, Tanko Abdulai, Miaomiao Niu, Zhicheng Luo, Yikang Wang, Ruiying Li, Fang Wang, Chongjian Wang, Zhenxing Mao

**Affiliations:** 1 Department of Epidemiology and Biostatistics, College of Public Health, Zhengzhou University, 100 Kexue Avenue, Zhengzhou, 450001 Henan PR China; 2 Department of Epidemiology, School of Public Health, Shanxi Medical University, Taiyuan, Shanxi PR China

**Keywords:** PSQI, Sleep quality, Anxiety symptoms, Rural population

## Abstract

**Background:**

The epidemiological evidence on the association of sleep quality on anxiety symptoms has been inconclusive. This study aimed to explore the association between sleep quality and anxiety symptoms in rural Chinese population and investigate whether age, lifestyles, and chronic diseases modified this association.

**Methods:**

A total of 27,911 participants aged 18–79 years from the Henan Rural Cohort Study were included in the study. Sleep quality was assessed using the Pittsburgh Sleep Quality Index (PSQI) scale. Poor sleep quality was defined as PSQI ≥6. Anxiety symptoms were evaluated with the two-item generalized anxiety disorder scale (GAD-2). Individual with score ≥ 3 was viewed as having anxiety symptoms. Logistic regression and restricted cubic spline were conducted to examine the association of sleep quality with anxiety symptoms.

**Results:**

Altogether, 6087 (21.80%) participants were poor sleepers and 1557 (5.58%) had anxiety symptoms. The odds of anxiety were increased with increment of PSQI score after fitting restricted cubic splines. The poor sleep quality was associated with a higher possibility of anxiety symptoms [odd ratio (OR): 4.60, 95% confidence interval (CI): 3.70–5.72] in men, and (OR: 3.56, 95% CI: 3.10–4.09) in women for multivariable analysis. Further, stratified analyses showed that the effect of sleep quality on anxiety symptoms could be modified by age, marital status, smoking status, drinking status, hypertension, and type 2 diabetes mellitus.

**Conclusions:**

A dose-response association between PSQI score and anxiety symptoms was found. In addition, the relationship between poor sleep quality and greater anxiety symptoms was observed in this rural population, especially in participants aged ≥60 years and those with unhealthy habits or had a chronic disease.

**Trial registration:**

The trial was prospectively registered on July 6, 2015 and available online at ClinicalTrials.gov ID: ChiCTR-OOC-15006699.

## Background

Anxiety, which is an emotion characterized by tension and restlessness, was associated with mental and physical discomfort [1]. The prevalence of anxiety disorders was globally up to 15% in the general population [2]. It was reported that generalized anxiety disorder (GAD) was present in 8.4% of adults from the Manaus Metropolitan Region [3]. As a type of psychological stress, anxiety will trigger a series of physiological events and cause a decrease in immunity [4]. Sufferers of anxiety can experience other physiological symptoms including fatigue, abdominal pain, headaches, dizziness, nausea, palpitations, and urinary incontinence [5]. Sleep, considered as a fundamental operating state of the central nervous system, may become one of the most important basic dimensions of brain function and mental health [6, 7]. Good sleep quality is important for optimal health status and wellness [8]. Previous researches suggested that better sleep quality could improve emotional well-being [9–11]. A web-based study showed a high prevalence of GAD and poor sleep quality in the Chinese public during COVID-19 outbreak [12]. Some studies have also reported an association between sleep quality and anxiety using Pittsburgh Sleep Quality Index (PSQI) [8, 13–16]. However, another study has shown that it is difficult to determine the cause and effect of sleep disturbance and anxiety [17].

Although studies have shown that poor sleep quality was associated with a higher prevalence of anxiety, these findings have been limited by study population and methodological variations, especially among under-developed rural populations in China [18]. China has a population of 1.4 billion, among which the rural population is large, accounting for 39.4% of the total population, according to the data from the China National Bureau of Statistics in 2019 [19]. The prevalence of mental disorders has been dramatic in China in recent decades [20]. And the prevalence of anxiety in rural China is higher than that in urban areas [21, 22]. Moreover, Henan is the most populous province with 48.3% of the rural population in 2018 [19]. And more than one fifth of the participants had poor sleep quality [23]. Focusing on people living in undeveloped region might be significant. Moreover, genetics showed that genes associated with circadian rhythms have been also related to a range of mental disorders [24]. The association between sleep quality and anxiety symptoms may provide references for the neurobiological mechanisms of mental disorders. In this context, to fill in the gap and add to the evidence for adverse effect of poor sleep quality on anxiety symptoms, this study was aimed at investigating the relationship between sleep quality assessed by PSQI and anxiety symptoms in Chinese rural population aged 18–79 years, and determined whether age, lifestyles and chronic diseases modified this association.

## Methods

### Study population

The participants of the current study were included from the Henan Rural Cohort, which was registered in Chinese Clinical Trial Register (Registration number: ChiCTR-OOC-15006699) and has been previously described in detail [25, 26]. Briefly, villagers aged 18–79 years were recruited from July 2015 to September 2017 by a multistage cluster sampling method from the local general population. Firstly, five rural counties in Henan province (central, south, north, east, and west) were chosen through simple cluster sampling on the basis of the local sufficient population source, support of the masses and local leadership, and medical conditions. Secondly, one to three rural townships of each county were selected by the local Centre for Disease Control and Prevention. Thirdly, the residents who gave a written informed consent were included as the study sample from each village of the selected township. Finally, a total of 39,259 participants (15,490 men and 23,769 women) who signed informed consent were included.

For the current analysis, a total of 29,995 participants completed the evaluation of anxiety symptoms. Furthermore, participants were excluded if they had missing data on PSQI score (*n* = 269). Because of the impaired sleep quality in shift workers [27, 28] and cancer [29], the participants who had self-reported experience of night shift work (*n* = 1530) or a history of cancer (*n* = 285) were further excluded to minimize the confounding bias. Finally, a total of 27,911 subjects were included in the present study.

Ethics approval was provided by the Zhengzhou University Life Science Ethics Committee, and signed informed consent was obtained for each participant. In addition, permission to administer each of the questionnaires, measures, or scales in the current study was obtained from every participant.

### Data collection

Data collection was performed by well-trained investigators in a face-to-face interview adopting a structured questionnaire. Demographic variables of participants included gender, age (continuous variable), marital status (married/cohabitation, other), educational levels (primary school or below, junior high school and senior high school or above), smoking status (non-smoker, or current smoker), alcohol consumption (non-drinker, or current drinker), and personal and family history of diseases.

Physical activity levels were classified into three categories: light, moderate and vigorous referenced to the criterion of the International Physical Activity Questionnaire [30]. Additionally, the anthropometric measurement was conducted on the basis of a standard protocol [31]. Height and weight were measured with individuals wearing light clothes and barefoot to the nearest 0.1 kg and 0.1 cm. Body mass index (BMI) was computed by body weight in kilograms divided by square of height in meters.

### Evaluation of sleep quality

Information on sleep was collected by PSQI [32], which consisted of 19 items. The scale that scores 0 to 21 has been widely used to evaluate sleep quality. A previous study reported that at least a cut-off PSQI score of 6 yields a sensitivity of 89.6% and a specificity of 86.5% [32]. Thus, participants with at least 6 PSQI score were considered as having a poor sleep quality in this study. Self-reported night sleep duration was obtained by asking the following question, “What time did you usually go to bed and wake up during the past month?” The sleep onset latency was assessed by the following question: “How long (in minutes) has it taken you to fall asleep each night during the past month?” The sleep initiation time was calculated as bed time plus sleep latency. The night sleep duration was computed on the basis of wake-up time and sleep initiation time [33].

### Definition of anxiety symptoms

The anxiety symptoms of participants were collected using the two-item generalized anxiety disorder scale (GAD-2) (feeling nervous, anxious, or on edge and not being able to stop or control worrying) yielding a sensitivity of 85% [34]. The scores of this scale ranged from 0 to 6. To examine the association between sleep quality and anxiety symptoms, this study dichotomized scores of the GAD-2 scale. Participants were defined as having anxiety symptoms if they scored ≥3 in the current study [35].

### Statistical analysis

Means ± standard deviations (SD) and frequencies (percentages) were presented for continuous and categorical variables, respectively. Multivariable restricted cubic regression spline curves [36] with 3 knots (5th, 50th, and 95th) were fitted to observe the association between continuous PSQI score and anxiety symptoms. Furthermore, the PSQI score was dichotomized to examine the association between poor sleep quality and anxiety symptoms with good sleep quality as reference group by performing logistic regression models. In the fully adjusted model, the potential confounders were adjusted according to the previous studies [14, 37], including age, gender, physical activity, marital status, smoking status, drinking status, educational levels, average monthly income, BMI, night sleep duration and napping duration. Considering the gender-specific prevalence of sleep quality, or anxiety, we investigated the associations stratified by gender through the full analyses [22, 23, 38]. Additionally, stratified analyses were conducted to examine whether the association between poor sleep quality and anxiety symptoms was potentially modified by age, gender, marital status, smoking status, drinking status, average monthly income, physical activity, BMI, snoring, hypertension, and type 2 diabetes mellitus(T2DM). Considering potential bias resulting from exclusion of participants, we did several sensitivity analyses to identify the association between sleep quality and anxiety symptoms by including subjects with shift working or cancer. A two-tailed *P* value of less than 0.05 was determined the statistical significance in the current study. All analyses were run on SAS version 9.3 (SAS Institute) and R version 3.5.1.

## Results

### Demographic characteristics

Table 1 displays the demographic characteristics of participants by anxiety symptoms. A total of 27,911 participants were included in this study. The mean (SD) age was 55.96 (12.22) years; 16,743 (59.99%) subjects were women; the mean (SD) PSQI score was 3.79 (2.73); 6087 (21.81%) individuals were poor sleepers; 1557 (5.58%) subjects have anxiety symptom. Those with anxiety symptoms were more likely to have lower education and income, lower physical activity levels, and have poorer sleep quality. In addition, the difference of demographic characteristics of participants between missing and non-missing information on PSQI score was reanalyzed. The findings implied that there was no difference except age, educational levels, and napping duration between two groups (See supplementary Table 1 in additional file 1). Likewise, the minor differences were found in both men and women. Thus, the missing data might be random and not affect the robustness of the current research.

**Table 1:** Demographic characteristics of participants according to anxiety symptoms stratified by gender

Variables	Total			Men			Women		
	No-anxiety	Anxiety	***P***	No-anxiety	Anxiety	***P***	No-anxiety	Anxiety	***P***
N	26,354	1557		10,719	449		15,635	1108	
Age (year), mean ± SD	55.94 ± 12.27	56.34 ± 11.37	0.180	57.21 ± 12.16	56.62 ± 12.05	0.316	55.07 ± 12.27	56.22 ± 11.09	< 0.001
Married/cohabitation, n (%)	23,697 (89.92)	1381 (88.70)	0.121	9645 (89.98)	397 (88.42)	0.282	14,052 (89.88)	984 (88.81)	0.257
Educational levels, n (%)			< 0.001			0.061			< 0.001
Primary school or below	11,921 (45.23)	848 (54.46)	.	3693 (34.45)	179 (39.87)	.	8228 (52.63)	669 (60.38)	.
Junior high school	10,131 (38.44)	547 (35.13)	.	4828 (45.04)	187 (41.65)	.	5303 (33.92)	360 (32.49)	.
Senior high school or above	4302 (16.32)	162 (10.40)	.	2198 (20.51)	83 (18.49)	.	2104 (13.46)	79 (7.13)	.
Average monthly income, n (%)			< 0.001			0.067			< 0.001
< 500 RMB	9654 (36.63)	693 (44.51)	.	4059 (37.87)	194 (43.21)	.	5595 (35.79)	499 (45.04)	.
500- RMB	8380 (31.80)	430 (27.62)	.	3285 (30.65)	122 (27.17)	.	5095 (32.59)	308 (27.80)	.
≥ 1000 RMB	8320 (31.57)	434 (27.87)	.	3375 (31.49)	133 (29.62)	.	4945 (31.63)	301 (27.17)	.
Current smoker, n (%)	5226 (19.83)	215 (13.81)	< 0.001	5182 (48.34)	212 (47.22)	0.639	44 (0.28)	3 (0.27)	0.948
Current drinker, n (%)	4515 (17.13)	174 (11.18)	< 0.001	4215 (39.32)	151 (33.63)	0.015	300 (1.92)	23 (2.08)	0.713
Physical activity, n (%)			0.018			0.872			0.002
Light	8353 (31.70)	440 (28.26)	.	3697 (34.49)	155 (34.52)	.	4656 (29.78)	285 (25.72)	.
Moderate	9672 (36.70)	601 (38.60)	.	2973 (27.74)	129 (28.73)	.	6699 (42.85)	472 (42.60)	.
Vigorous	8329 (31.60)	516 (33.14)	.	4049 (37.77)	165 (36.75)	.	4280 (27.37)	351 (31.68)	.
BMI (kg/m^2^), mean ± SD	24.74 ± 3.57	24.31 ± 3.58	< 0.001	24.45 ± 3.45	23.90 ± 3.40	< 0.001	24.93 ± 3.64	24.48 ± 3.63	< 0.001
Night sleep duration(h), mean ± SD	7.72 ± 1.26	7.47 ± 1.51	< 0.001	7.72 ± 1.27	7.57 ± 1.50	0.041	7.73 ± 1.26	7.43 ± 1.52	< 0.001
Napping duration (min), mean ± SD	58.84 ± 50.76	56.69 ± 53.20	0.121	64.10 ± 50.20	62.12 ± 52.52	0.412	55.23 ± 50.83	54.49 ± 53.34	0.656
PSQI score, mean ± SD	3.66 ± 2.61	6.04 ± 3.63	< 0.001	3.22 ± 2.27	5.20 ± 3.21	< 0.001	3.96 ± 2.77	6.38 ± 3.74	< 0.001
Poor sleep quality, n (%)	5315 (20.17)	772 (49.58)	< 0.001	1556 (14.52)	185 (41.20)	< 0.001	3759 (24.04)	587 (52.98)	< 0.001

Abbreviation: *SD* Standard deviation, *BMI* Body mass index, *PSQI* Pittsburgh Sleep Quality Index

### Dose-response association between PSQI score and anxiety symptoms

**Fig. 1:**
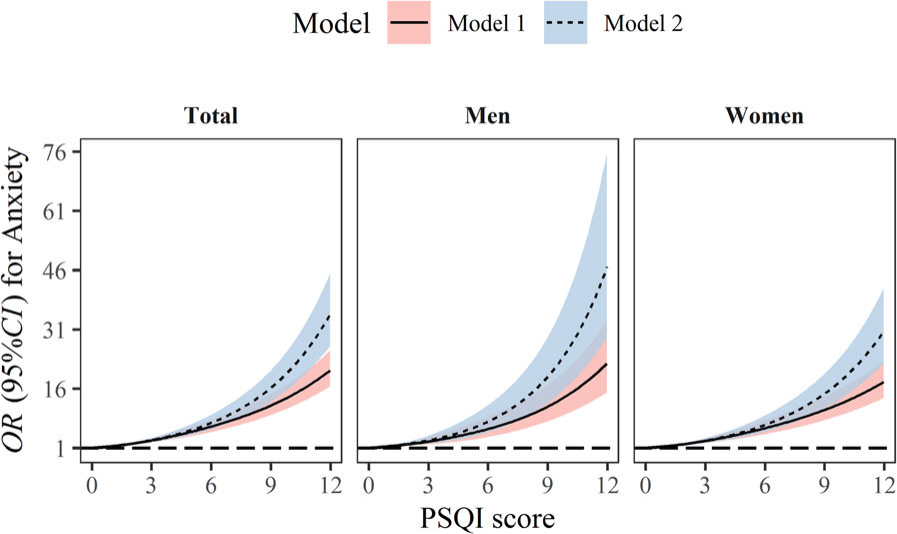
Restricted cubic spline plot of elevated trend of Pittsburgh Sleep Quality Index (PSQI) score with anxiety symptoms stratified by gender. Model 1: unadjusted; Model 2: adjusted for age, gender (only in total population), physical activity, marital status, smoking status, drinking status, educational levels, average monthly income, body mass index (BMI), night sleep duration and napping duration

Figure 1 presents the association between continuous PSQI score and the prevalence of anxiety symptoms. There is an increased likelihood of anxiety symptoms with the elevated PSQI score for crude model in total population. After additional adjustment for age, gender, physical activity, marital status, smoking status, drinking status, educational levels, average monthly income, BMI, night sleep duration, and napping duration, the association appeared to be slightly enhanced and remained to be significant. Similar findings were available in men and women.

### Association between poor sleep quality and anxiety symptoms

Table 2 reports results of sleep quality and anxiety symptoms, with less than 6 scores of PSQI as reference category. Compared to the reference group, poor sleep quality (PSQI ≥6) was associated with a higher possibility of anxiety symptoms [odd ratio (OR): 3.85, 95% confidence interval (CI): 3.42–4.33] in total populations, (OR: 4.60, 95%CI: 3.70–5.72) in men, and (OR: 3.56, 95%CI: 3.10–4.09) in women on multivariable analysis.

**Table 2:** OR (95% CI) of sleep quality and anxiety symptoms stratified by gender

Sleep quality	Cases/N	Model 1	Model 2
Total			
Good	785/21,824	1	1
Poor	772/6087	3.89 (3.51–4.32)	3.85 (3.42–4.33)
Men			
Good	264/9427	1	1
Poor	185/1741	4.13 (3.39–5.02)	4.60 (3.70–5.72)
Women			
Good	521/12,397	1	1
Poor	587/4346	3.56 (3.15–4.03)	3.56 (3.10–4.09)

Model 1: unadjusted;

Model 2: adjusted for age, gender, physical activity, marital status, smoking status, drinking status, educational levels, average monthly income, body mass index (BMI), night sleep duration and napping duration

### Stratified analysis for poor sleep quality and anxiety symptoms

**Fig. 2:**
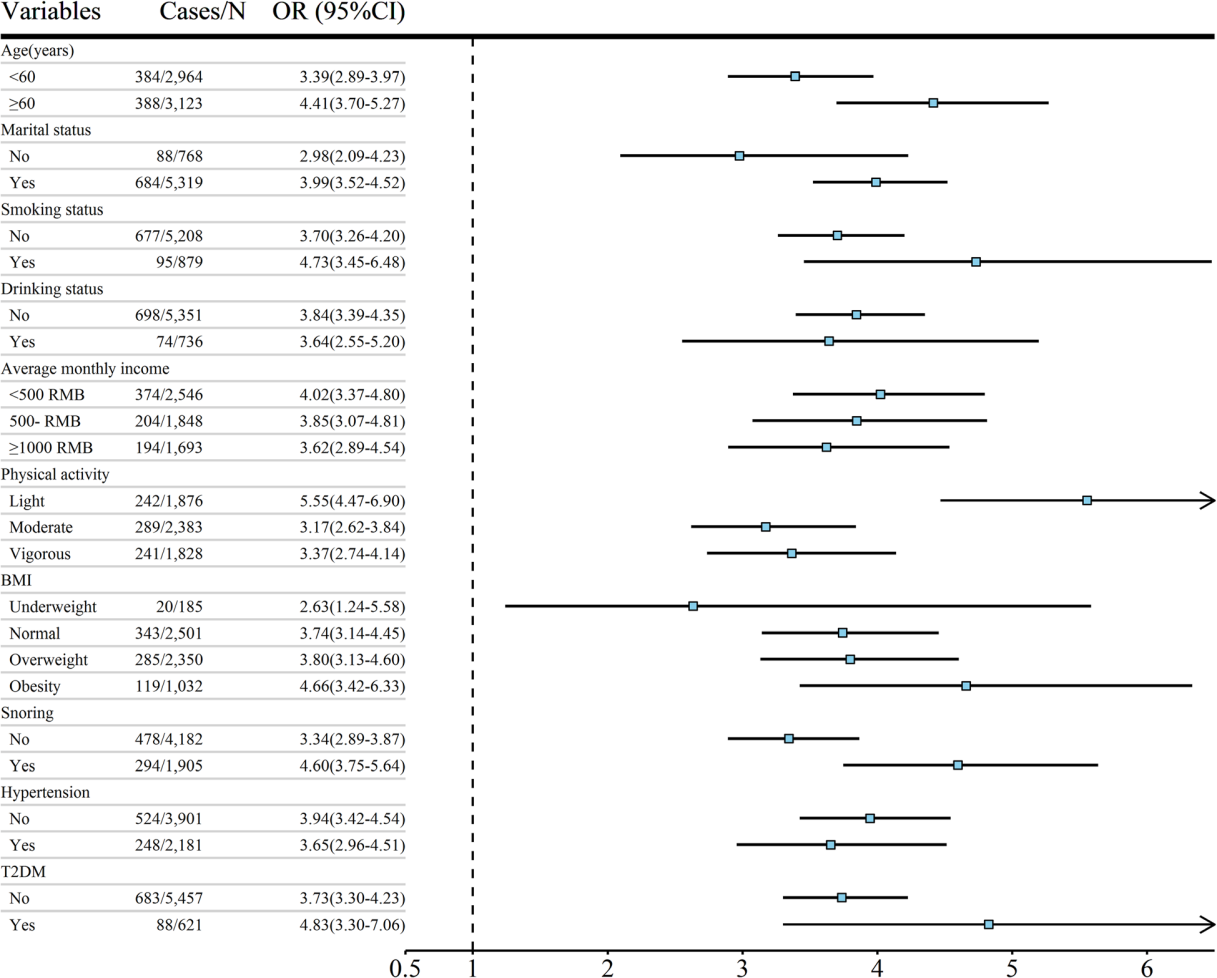
OR (95% CI) of poor sleep quality (PSQI ≥ 6) between anxiety symptoms stratified by potential modifiers. Adjusted for age, gender (only in total population), physical activity, marital status, smoking status, drinking status, educational levels, average monthly income, body mass index (BMI), night sleep duration and napping duration. Abbreviation: PSQI, Pittsburgh Sleep Quality Index

The results of the stratified analysis for anxiety symptoms are shown in Fig. 2. Anxiety symptoms was associated with poor sleep quality among aged 60 or older (OR: 4.41, 95% CI: 3.70–5.27), married (OR: 3.99, 95% CI: 3.52–4.52), smokers (OR: 4.73, 95% CI: 3.45–6.48), participants with light level of physical activity (OR: 5.55, 95% CI: 4.47–6.90), obesity (OR: 4.66, 95% CI: 3.42–6.33), snoring (OR: 4.60, 95% CI: 3.75–5.64), T2DM (OR: 4.83, 95% CI: 3.30–7.06) (Fig. 2).

### Sensitivity analysis

In sensitivity analyses, similar results were observed when we included the participants with shift working (See supplementary Table 2 in additional file 1). Comparing participants with poor sleep, the ORs were 3.86 (95% CI: 3.44–4.33) for total, 4.82 (95% CI: 3.90–5.95) for men, and 3.49(95% CI: 3.04–4.00). When the participants with cancer were included, the results did not change materially (See supplementary Table 3 in additional file 1).

## Discussion

The current study was the first to focus on the association between poor sleep quality and the odds of anxiety symptoms in a large Chinese rural population. The results of this study demonstrated that a positive association between poor sleep quality and anxiety symptoms was significant in both men and women among a large Chinese rural population. After the stratified analysis, stronger positive associations were observed among individuals aged 60 or above, smokers, and individuals with light level of physical activity, obesity and T2DM.

China is a country with 1.4 billion people and 39.4% of Chinese people live in rural areas [19]. According to the newest data from China National Bureau of Statistics in 2018, the rural population of Henan were higher than the national level, accounting for 48.3% of the total population of the whole province [19]. However, in rural areas, most people with poor sleep quality were either not treated or treated inadequately. There is an obvious gap between the levels of urban and rural health and the health level of rural residents is relatively low [39]. A previous study showed that the inequality and imbalance of medical facilities were found across 203,801 villages and 1609 townships in Henan province [40]. To our knowledge, there is still the lack of evidence on association between the sleep quality and anxiety symptoms in rural regions. Therefore, this study is meaningful among rural population. The study found that those with anxiety symptoms were more likely to have a lower income and be exposed to unhealthy lifestyles, such as lower level of physical activity.

This study presented the association between poor sleep quality and anxiety symptoms, which is consistent with a previous study that poor sleep quality was strongly associated with anxiety symptoms among women [41]. Several previous studies on patients with coronary artery bypass graft identified that a better sleep quality was related to a lower anxiety level [9, 42]. Some studies have also reported an association between sleep quality and anxiety symptoms using PSQI [8, 13–16]. For example, one of the studies found that patients with an increase in preoperative state anxiety had a 18.6% higher odds of prevalent poor sleep quality (95% CI: 1.074 to 1.115), after controlling the potential confounders [8]. Consistent with previous studies, our study found that poor sleep quality was associated with a higher possibility of anxiety symptoms (OR: 3.85, 95% CI: 3.42–4.33) in total population. Although previous studies have reported an association between poor sleep quality and anxiety symptoms, these studies were limited to specific populations (adult women [13], older Chinese [14], cardiovascular patient [15], after coronary artery bypass surgery [8], T2DM [16]). Few studies have been sufficiently large to show a statistically significant modifying effect of sleep quality and anxiety in an overall healthy population. However, another study found that poor sleep quality was associated with both depression and anxiety, whereas only daytime sleepiness was associated with anxiety symptoms in older adults [43].

The mechanisms behind the association between sleep quality and anxiety remain unclear. Moreover, lack of sleep can bring a range of adverse neurobehavioral outcomes and physiological changes, such as inattention, depression, impaired glucose tolerance, and sympathetic nervous system activation [44]. These changes in sleep quality may manifest as the onset of mental illness, including anxiety. Nevertheless, these findings based on cross-sectional data, are limited to confirming a causal relationship between sleep quality and anxiety, and the exact mechanisms are needed to be studied.

The study suggests that the government should strengthen public education, use mass media to actively publicize the need for exercise and guide them on how to carry out appropriate activities. At the same time, epidemiologists should focus on the identification and early intervention of the elderly population over 60 years old. In the family, relatives should pay attention to the sleep quality of family members, develop good sleep habits, so as to reduce the occurrence of anxiety symptoms. Future prospective studies should examine multiple facets of sleep quality with the aim of better characterizing sleep quality and improving treatments.

This study has the following strengths. First, poor sleep quality is a symptom of many health problems, such as anxiety symptoms, hypertension [26], overweight/obesity [45], coronary heart disease [46] and so on. This study thoroughly clarified the association between poor sleep quality and anxiety symptoms in a large-scale rural population from the Henan rural cohort study. Second, this is the first analysis of this association in rural China so far. It gave us a chance to understand the relationship between sleep quality and anxiety symptoms in the Chinese rural population.

The current study also has some limitations. First, this was a cross-sectional study, and there is the possibility of reverse causality. Long-term longitudinal studies are recommended to characterize the overall changes in sleep quality and anxiety symptoms. Second, although the PSQI is a well-validated scale of sleep quality, the recall bias on the results cannot be inevitable thoroughly. Third, we did not consider the factors such as living arrangements, or necessary medical treatments which might impact sleep, or anxiety symptoms. Finally, the population is not nationally representative, therefore, the extrapolation of the results may be limited.

## Conclusions

A dose-response association between PSQI score and increased odds of anxiety symptoms was observed. Moreover, this study also found that poor sleep quality contributed to the increased prevalence of anxiety symptoms in a Chinese rural population, especially in those who were 60 years old or above and smokers, as well as those with light level of physical activity and obesity. In addition, these findings suggest that people should develop good sleeping habit and reduce occurrence of poor sleep quality to furthermore prevent anxiety symptoms.

## Supplementary information

**Additional file 1: Table S1.** Difference of demographic characteristics of participants with between missing and non-missing on PSQI score stratified by gender. **Table S2.** OR (95% CI) of sleep quality and anxiety symptoms included participants with shift working stratified by gender. **Table S3.** OR (95% CI) of sleep quality and anxiety symptoms included participants with shift workers stratified by gender.

## Data Availability

The data used in this study are available and will be provided by the corresponding author on a reasonable request.

## Abbreviations

PSQI: Pittsburgh Sleep Quality Index
GAD: Generalized anxiety disorder
BMI: Body mass index
GAD-2: The two-item generalized anxiety disorder scale
SD: Standard deviation
T2DM: Type 2 diabetes mellitus
