# Supplementary material for: Dose-response association of sleep quality with anxiety symptoms in Chinese rural population: the Henan rural cohort
Source: BMC Public Health. 2020 Aug 27;20:1297. doi: 10.1186/s12889-020-09400-2 (PMC7450150; doi:10.1186/s12889-020-09400-2)
Supplement: Supplementary file 1 — Additional file 1: Table S1. Difference of demographic characteristics of participants with between missing and non-missing on PSQI score stratified by gender. Table S2. OR (95% CI) of sleep quality and anxiety symptoms included participants with shift working stratified by gender. Table S3. OR (95% CI) of sleep quality and anxiety symptoms included participants with shift workers stratified by gender. [file 12889_2020_9400_MOESM1_ESM.docx]

Supplementary Table 1. Difference of demographic characteristics of participants with between missing and non-missing on PSQI score stratified by gender

| Variables | Total | | | Men | | | Women | | |
| --- | --- | --- | --- | --- | --- | --- | --- | --- | --- |
|  | Non-missing on PSQI score | Missing on PSQI score | ***P*** | Non-missing on PSQI score | Missing on PSQI score | ***P*** | Non-missing on PSQI score | Missing on PSQI score | ***P*** |
| N | 29726 | 269 |  | 12135 | 99 |  | 17591 | 170 |  |
| Age(year), mean±SD | 55.44±12.37 | 56.96±11.81 | 0.045 | 56.47±12.39 | 58.55±11.56 | 0.097 | 54.72±12.30 | 56.03±11.89 | 0.168 |
| Married/cohabitation, n (%) | 26801(90.16) | 242(89.96) | 0.914 | 10951(90.24) | 90(90.91) | 0.824 | 15850(90.10) | 152(89.41) | 0.764 |
| Educational levels, n (%) |  |  | 0.006 |  |  | 0.212 |  |  | 0.031 |
| Primary school or below | 13133(44.18) | 144(53.53) | . | 4050(33.37) | 39(39.39) | . | 9083(51.63) | 105(61.76) | . |
| Junior high school | 11592(39.00) | 82(30.48) | . | 5480(45.16) | 36(36.36) | . | 6112(34.75) | 46(27.06) | . |
| Senior high school or above | 5001(16.82) | 43(15.99) | . | 2605(21.47) | 24(24.24) | . | 2396(13.62) | 19(11.18) | . |
| Average income per month, n (%) |  |  | 0.145 |  |  | 0.407 |  |  | 0.130 |
| <500 RMB | 10741(36.13) | 91(33.83) | . | 4444(36.62) | 30(30.30) | . | 6297(35.80) | 61(35.88) | . |
| 500- RMB | 9371(31.52) | 76(28.25) | . | 3713(30.60) | 32(32.32) | . | 5658(32.16) | 44(25.88) | . |
| ≥1000 RMB | 9614(32.34) | 102(37.92) | . | 3978(32.78) | 37(37.37) | . | 5636(32.04) | 65(38.24) | . |
| Current smoker, n (%) | 5972(20.09) | 49(18.22) | 0.445 | 5923(48.81) | 46(46.46) | 0.642 | 49(0.28) | 3(1.76) | <0.001 |
| Current drinker, n (%) | 5192(17.47) | 38(14.13) | 0.151 | 4848(39.95) | 37(37.37) | 0.602 | 344(1.96) | 1(0.59) | 0.199 |
| High vegetables and fruits intake, n (%) | 14232(47.88) | 130(48.51) | 0.838 | 5805(47.84) | 53(54.08) | 0.218 | 8427(47.91) | 77(45.29) | 0.498 |
| High fat diet, n (%) | 5387(18.12) | 57(21.19) | 0.194 | 2866(23.62) | 34(34.34) | 0.012 | 2521(14.33) | 23(13.53) | 0.767 |
| Physical activity, n (%) |  |  | 0.351 |  |  | 0.044 |  |  | 0.167 |
| Light | 9479(31.89) | 83(30.86) | . | 4201(34.62) | 42(42.42) | . | 5278(30.00) | 41(24.12) | . |
| Moderate | 10940(36.80) | 91(33.83) | . | 3419(28.17) | 17(17.17) | . | 7521(42.75) | 74(43.53) | . |
| Vigorous | 9307(31.31) | 95(35.32) | . | 4515(37.21) | 40(40.40) | . | 4792(27.24) | 55(32.35) | . |
| BMI(kg/m^2^), mean±SD | 24.74±3.57 | 24.42±3.41 | 0.151 | 24.50±3.47 | 23.76±3.30 | 0.035 | 24.90±3.63 | 24.81±3.42 | 0.742 |
| Night sleep duration(h), mean±SD | 7.71±1.30 | 7.75±1.40 | 0.639 | 7.70±1.32 | 7.78±1.42 | 0.594 | 7.71±1.29 | 7.73±1.40 | 0.859 |
| Napping duration(min), mean±SD  Sletim | 59.04±51.61 | 47.06±49.08 | <0.001 | 64.17±51.38 | 59.64±53.63 | 0.385 | 55.50±51.47 | 39.76±44.80 | <0.001 |

Abbreviation: SD, standard deviation; BMI, body mass index; PSQI, Pittsburgh Sleep Quality Index

Supplementary Table 2. OR (95% CI) of sleep quality and anxiety symptoms included participants with shift working stratified by gender

| **Sleep quality** | **Cases/N** | **Model 1** | **Model 2** |
| --- | --- | --- | --- |
| Total |  |  |  |
| Good | 812/23111 | 1 | 1 |
| Poor | 807/6394 | 3.95(3.57-4.38) | 3.86(3.44-4.33) |
| Men |  |  |  |
| Good | 272/10185 | 1 | 1 |
| Poor | 199/1880 | 4.31(3.57-5.22) | 4.82(3.90-5.95) |
| Women |  |  |  |
| Good | 540/12926 | 1 | 1 |
| Poor | 596/4440 | 3.56(3.15-4.02) | 3.49(3.04-4.00) |

Model 1: unadjusted;

Model 2: adjusted for age, gender, physical activity, marital status, smoking status, drinking status, educational levels, average monthly income, body mass index (BMI), night sleep duration and napping duration

Supplementary Table 3. OR (95% CI) of sleep quality and anxiety symptoms included participants with shift workers stratified by gender

| **Sleep quality** | **Cases/N** | **Model 1** | **Model 2** |
| --- | --- | --- | --- |
| Total |  |  |  |
| Good | 795/22037 | 1 | 1 |
| Poor | 784/6159 | 3.95(3.57-4.38) | 3.82(3.40-4.29) |
| Men |  |  |  |
| Good | 265/9483 | 1 | 1 |
| Poor | 186/1750 | 4.31(3.57-5.22) | 4.59(3.69-5.70) |
| Women |  |  |  |
| Good | 530/12554 | 1 | 1 |
| Poor | 598/4409 | 3.56(3.15-4.02) | 3.53(3.08-4.05) |

Model 1: unadjusted;

Model 2: adjusted for age, gender, physical activity, marital status, smoking status, drinking status, educational levels, average monthly income, body mass index (BMI), night sleep duration and napping duration
